# Benjamin Franklin

**Published:** 1870-04

**Authors:** 


### BENJAMIN FRANKLIN.

WITHIN a few days we listened to a lecture on the life of Franklin, delivered by Judge Vanvorst to the 23d St. Public School. There were perhaps a thousand boys present, who gave their most undivided attention, and doubtless impressions were made by that lecture, which will aid to mould for years the character of many of those who were present. Judge Vanvorst has been a steadfast friend to the public school system, and for many years has given his time and influence and counsels in this direction, and if his admirable and impressive lecture could be delivered to every one of the public schools in

New York city, male and female, a great good would be done.

